# Evaluating the programme and behavior change theories of a community alcohol education intervention in rural Sri Lanka: a study protocol

**DOI:** 10.1080/16549716.2023.2273625

**Published:** 2023-11-16

**Authors:** Jane Brandt Sørensen, K. S. Kylie Lee, Andrew Dawson, Angela Dawson, Lalith Senarathna, P. H. G. Janaka Pushpakumara, Thilini Rajapakse, Flemming Konradsen, Nick Glozier, Katherine M. Conigrave, Prabash Siriwardhana, David Hansen, Alexandra Buhl, Chamill Priyadhasana, Kamal Senawirathna, Malith Herath, Sudesh Mantillake, Priyantha Fonseka, Melissa Pearson

**Affiliations:** a Department of Public Health, University of Copenhagen, Copenhagen, Denmark; b NHMRC Centre of Research Excellence in Indigenous Health and Alcohol, Discipline of Addiction Medicine, Faculty of Medicine and Health, The University of Sydney, Camperdown, NSW, Australia; c The Edith Collins Centre (Translational Research in Alcohol Drugs and Toxicology), Royal Price Alfred Hospital, Camperdown, NSW, Australia; d Centre for Alcohol Policy Research, La Trobe University, Bundoora, VIC, Australia; e National Drug Research Institute, Faculty of Health Sciences, Curtin University, Perth, WA, Australia; f Burnet Institute, Melbourne, Victoria, Australia; g Faculty of Medicine, South Asian Clinical Toxicology Collaboration, University of Peradeniya, Peradeniya, Sri Lanka; h School of Public Health, University of Technology Sydney, Sydney, Australia; i Faculty of Applied Sciences, Rajarata University of Sri Lanka, Mihintale, Sri Lanka; j Department of Family Medicine, Faculty of Medicine and Allied Sciences, Rajarata University of Sri Lanka, Saliyapura, Sri Lanka; k Department of Psychiatry, University of Peradeniya, Peradeniya, Sri Lanka; l Central Clinical School, Faculty of Medicine and Health, University of Sydney, Sydney, Australia; m ARC Centre of Excellence for Children and Families over the Life Course, Sydney, Australia; n Royal Prince Alfred Hospital, Drug Health Services, Sydney, NSW, Australia; o Faculty of Medicine and Health, Central Clinical School, Camperdown, NSW, Australia; p Department of Sociology, Rajarata University of Sri Lanka, Mihintale, Sri Lanka; q Filmmaker, The Perfect World, Sydney, Australia; r Faculty of Arts, University of Peradeniya, Peradeniya, Sri Lanka; s Central Clinical School, University of Sydney, Sydney, Australia

**Keywords:** Alcohol, community-based, health promotion, behaviour change wheel, education entertainment

## Abstract

Risky alcohol use is a major public health problem globally and in Sri Lanka. While a reduction in alcohol consumption can result in physical, mental, and social benefits, behaviour change is difficult to achieve. Effective, context-adapted interventions are required to minimise alcohol-related harm at a community level. THEATRE is a complex, community-based intervention evaluating whether a promising Sri Lankan pilot study that utilised arts-based research to moderate alcohol use can be scaled up. While the scaled-up pilot study protocol is presented elsewhere, the aim of this protocol paper is to describe the intervention programme theory and evaluation design, and modifications made to the study resulting from COVID-19 and the financial crisis. Drawing on the Behaviour Change Wheel (BCW) and Theoretical Domains Framework, behaviour change theories are presented with potential pathways to guide implementation and evaluation. Alcohol consumption patterns and context of drinking is detailed. The multifaceted intervention targets individuals and communities using arts-based interventions. Four of nine BCW functions are employed in the design of the intervention: education, persuasion, modelling and enablement, and training. Modifications made to the study due to COVID-19 and the financial crisis are described. Ethical approval was obtained from the Ethics Review Committee, Faculty of Medicine and Allied Sciences, Rajarata University of Sri Lanka (ERC/2018/21—July 2018 and Feb 2022) and the University of Sydney (2019/006). Findings will be disseminated locally to community members and key stakeholders and via international peer-reviewed publications.

## Introduction

Alcohol use contributes to 1.78 million deaths annually worldwide (2020) and constitutes the leading risk factor for men aged 15–49 years [1]. Here risky consumption refers to drinking that poses a risk of harm, or is already causing harm, to the individual or to others. This can include alcohol use disorders. Accordingly, reducing risky drinking is included in the Sustainable Development Goals (SDG Health Target 3.5) [2]. However, while there have been reductions in alcohol consumption in high-income countries (HIC), this has not been the case in low- and middle-income countries (LMICs) where alcohol consumption is on the increase in many countries [3].

In the middle-income country of Sri Lanka alcohol rates are also rising [4]. Alcohol consumption is generally under-reported in Sri Lanka, likely because it can be considered immoral in a country where Buddhism is the predominant religion [5]. The homebrew and illicit alcohol ‘kasippu’ is commonly used, especially in rural areas, accounting for 40% of total alcohol consumption (between 2008 and 2010) [6]. Many Sri Lankans abstain from drinking, especially women [7,8], and those who do consume alcohol do so at much higher rates than the average. Risky alcohol use among Sri Lankan men has been linked to self-medication to cope with physical, mental, and financial stress, or to counter boredom [9]. As in other settings, risky alcohol use has been found to lead to cirrhosis [10], depression [11], self-harm [12,13] injury through traffic accidents [14], and it is linked to social issues including poverty [15], household conflict [13], and domestic violence [16]. Children are particularly vulnerable to risky alcohol use in the household, for example through unintentional injuries [17], child abuse [18] neglect or difficulties keeping up in school [19]. Alcohol consumption in rural Sri Lanka has been found to be a habitual, social behaviour that helps reinforce social connections [20]. Previous studies have demonstrated that men ‘must’ consume alcohol, that ‘men cannot help it’, and that there is no way back from heavy drinking [20,21]. A recent qualitative study on patterns of drinking found that alcohol consumption in rural Sri Lanka is an important social activity among certain groups of men and unwritten guiding principles dictated that ‘moderate consumption’ in social settings was appropriate [20]. Furthermore, studies have explored the notion of a masculine social identity tied to drinking alcohol [20,21]. Alcohol consumption is thus a social practice often linked to peer pressure and status and is believed to form social and economic connections [20]. Inappropriate behaviour during and after drinking occasions can be excused by non-drinkers as an unfortunate norm [20]. While many men may not be aware of the consequences of risky drinking to themselves, their families, and the community, it is often associated with shame and embarrassment to the wider family [13]. Often, affected family members in rural Sri Lanka have limited access to assistance as alcohol treatment or support services are scarce [22].

Sri Lankan government policy includes a commitment to reduce alcohol use [23]. This entails a ban on alcohol advertisement in sports; the introduction of minimum purchasing age; restrictions on alcohol sales on certain holidays; and increased taxes on alcohol [20].

The complex nature in which alcohol is consumed in many LMICs, including Sri Lanka, raises several issues for research. While alcohol research in HICs has primarily focused on market-based sources, i.e. formal outlets, alcohol research and interventions in LMICs also need to consider a wider context of drinking. Furthermore, prevention and treatment options differ vastly between HICs and LMICs [3].

Our pilot study found that direct collaboration with communities through a participatory theatre approach gave affected communities the skills, direction, and confidence to make sustained changes in reducing alcohol use and associated harms, and improving mental health [24]. Participatory theatre was used in this pilot to suit the Sri Lankan context where narrative forms of communication are popular (see for example [25]). Here, storytelling is used to make sense of the world, understand events and recount experiences [26]. Such an arts-based approach to research and change that infuses media stories with health information is commonly referred to as Education-Entertainment (EE) [27]. Compared to other approaches to changing health risk behaviours, for example, ‘Behaviour Change Communication’ (BCC) and ‘Communication for Development’ (C4D), EE entails an important persuasive effect [28] to help increase health literacy and communicate health information [29]. EE messages have been shown to foster involvement in the storyline [29], which can create an emotional experience of being part of the narrative and help to identify with the characters [27]. Research shows that an EE approach is especially valuable where issues are complex, and contentious or where appeals to logic or reason may not be well suited [30]. Thus, the narrative forms used in EE may be impactful because they help people process new or difficult information, produce stronger responses in attitudes and intentions, and provide connections to role models [27,31].

While the results from the pilot study were promising, they were conducted in only one village with a comparison control village. More robust research was needed to thoroughly assess effectiveness and sustainability over time. Therefore, a scaled-up pilot study (THEATRE) was devised to explore whether a community-based alcohol education programme can reduce alcohol use and related harms in rural Sri Lankan villages. We originally designed the THEATRE study as an effectiveness trial, but changes to the design were needed due to a series of crises – the protocol for this can be found elsewhere [32]. This protocol emphasises the theoretical basis of how the intervention was developed. As such, this companion paper will enable us to obtain a deeper understanding of the ‘active ingredients’ (the how and why things work) of the intervention [33]. Since complex interventions should be informed by evidence, context, and theory [34], this paper has two objectives: (i) To present our behaviour change theories and outline our hypothesis for the pathways to behaviour change, including the processes and steps taken by the research team to develop the arts-based intervention and its accompanying evaluation; and (ii) to document the steps undertaken to adapt the intervention to the COVID-19 pandemic and following the Sri Lanka financial crisis.

## Methods and analysis

### Setting and context

This intervention is conducted in 15 villages in the North Central Province of Sri Lanka (+10,000 km^2^). In villages in this rural area, the majority of the population are Sinhalese and Buddhist. Many families make a living through agriculture. Income is often irregular, earned with casual daily wages. Rapid changes in employment opportunities in semi-urban villages have led younger individuals to seek non-agricultural employment opportunities [35]. National and international migration for work is common. High levels of self-harm have been recorded in the North Central Province [36], which have repeatedly been linked to alcohol consumption [12]. In this setting, community acceptable behaviour is associated with norms of morality, respectability, and fear of public shame [37]. Obedience towards individuals above oneself in the social hierarchy is crucial and influenced by gender and age [38].

Sri Lanka has faced several challenges in recent years. On 21 April 2019, a terrorist attack resulted in more than 270 deaths. This led to a nationwide curfew and prolonged bans on large gatherings. As life was returning to normal, the first cases of COVID-19 were detected in Sri Lanka in January 2020. In response, the Sri Lankan government imposed a range of social distancing measures, which changed the social context in the study area (e.g. bans on community gatherings and night-time activities, and the sales of alcohol). Since 2021, Sri Lanka has faced a financial crisis that severely affected access to basic goods such as fuel, food, and medicine.

To thoroughly understand the environment in which this research is framed, we mapped the alcohol environment using the recommendations by Walls et al. (Appendix 1) [39]. Here, the alcohol supply chain, alcohol acquisition, context of drinking, and subsequent outcomes were assessed to identify important points of intervention. In developing the intervention, we especially emphasized the main drivers of risky alcohol drinking (i.e. expectations for men to consume and self-medication) and the root causes of the harmful effects of risky alcohol drinking (i.e. a gendered hierarchical society, socio-economic difficulties, and alcohol bringing shame to the household).

### Study team

The study is a multi-disciplinary collaboration between several universities and institutions that brings together experts in public health, addiction medicine, social science, toxicology, and fine arts. The study team has expertise in alcohol prevention and treatment, community-based research, devising community-based narrative arts projects with health messages, and community-based interventions in LMICs. Many of the research team members have worked at the community level in Sri Lanka for decades on issues related to alcohol, self-harm, suicide, and social capital.

### Patient and public involvement

This complex intervention, including its materials, was initially developed with community members in the pilot village [24]. Following COVID-19 and the financial crisis, the study intervention was adjusted in consultation with selected community members to ensure appropriateness. Community mobilisation is thus a key feature of this intervention. Local advocates work alongside the research team to disseminate materials and findings.

### Conceptual framework and programme theory

**Figure 1.**
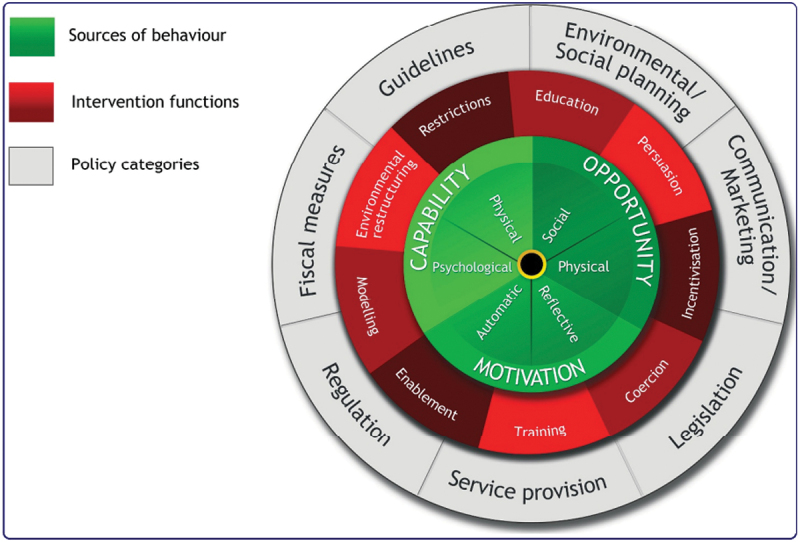
The COM-B model made up of the three components needed for behaviour (B) change: capability (C), opportunity (O) and motivation (M), which is linked to interventions and policies [47].

For this study we made use of the GUIDED and TidieR to describe the intervention development process (Annex 4 and 5). The UK Medical Research Council’s guidelines for complex interventions highlight the importance of theory to inform health behaviour change interventions [34]. The Behaviour Change Wheel (BCW) and the Theoretical Domains Framework (TDF) provide a comprehensive framework for factors likely to influence behaviour, as well as a theoretically informed development of interventions that promote improved health outcomes. Developed by Michie et al., the BCW is a framework that captures several behaviour change theories in one [40] (see Figure 1). The inner circle comprises the COM-B model, which is made up of three components needed for *behaviour* (B) change: physical and psychological *capability* (C), social and environmental *opportunity* (O), and *motivation* (M), which can be reflective or automatic. The middle ring links behaviour to interventions and policies, comprising nine interventions, and the outer ring has seven policy-level strategies.

**Figure 2.**
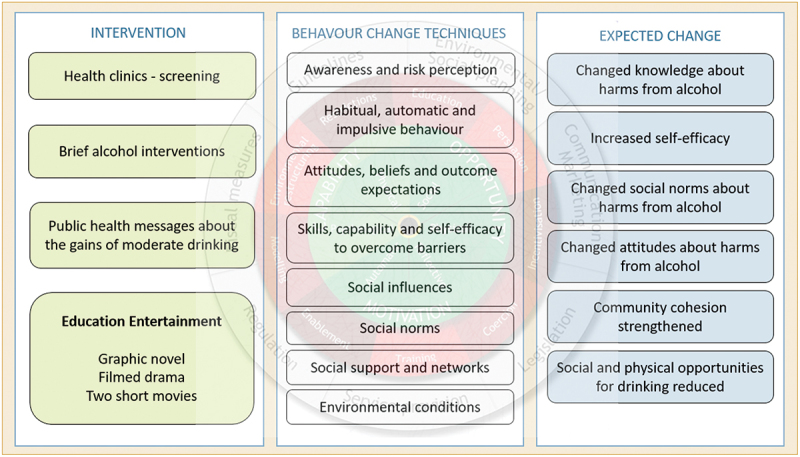
The THEATRE intervention components, behaviour change techniques that address the intervention functions and expected change.

The programme theory for this study was based on BCW and TDF to capture the complexity of the intervention (Figure 2), similar to other intervention development research [41]. Unpacking the three categories in the COM-B (Capability, Opportunity and Motivation), TDF consists of 14 components that categorise influences on behaviour (such as ‘Knowledge’, ‘Skills’, and ‘Beliefs about Capabilities’).

#### Intervention design and adaptation

To create change in health behaviour, an intervention should be multifaceted and target different individuals and the community using a range of elements [40]. With a foundation in our contextual understanding of alcohol consumption in rural Sri Lanka outlined in Appendix 1, the THEATRE intervention targets the community at four levels: individuals engaging in risky alcohol drinking; treatment access; support to family members affected by the drinking of others; and community mobilisation. Further, four functions of the intervention are employed in its design to moderate alcohol consumption: education, persuasion, modelling and enablement, and training. The key focus areas used to obtain alcohol behaviour change were identified using the BCW. Specifically, our aim is to: increase knowledge and awareness about harmful alcohol use; model healthy drinking behaviour and recovery practice to increase self-efficacy; improve awareness of care options; reduce alcohol-related harms for individuals and families; challenge attitudes and social norms that support risky alcohol use; and empower communities to provide a supportive social and physical environment to address risky drinking, with the overall aim of reducing the prevalence of harmful alcohol use – the primary study outcome.

### Changing delivery of the intervention due to multiple crises

Due to the multiple crises experienced in Sri Lanka from 2019, parts of the intervention were difficult or impossible to implement. Using the framework for reporting adaptions and modifications to evidence-based interventions (FRAME) [42], we modified the intervention to fit the current context, to ensure the intervention could be delivered in a manner compliant with COVID-19 restrictions and to reach community members without undermining their or the research staff’s safety (Appendix 2). Especially, the face-to-face intervention components needed to be adapted, since they were intended to be delivered in person in clinics and village gatherings, contravening social distancing regulations. An overview of the different intervention components, the rationale for including them, as well as how they were adapted is provided below.

#### Education entertainment (EE)

A major aspect of the intervention in the pilot study [24] was a series of EE components. During the pilot, street dramas were created and presented in close collaboration with community members [24]. In 2013 and 2020/2021, a collaborative, community-devised model was used to create story outlines that presented locally relevant information about the impacts of risky drinking at individual, family and societal level and tips to address these. Identification, transportation, and emotion are considered to be the most important elements of EE [31], and so collaboration with an experienced filmmaker (DH) and Fine Arts academics were crucial to guide the collaborative and creative process, and embedding the health-related content [43]. Each component involved community members as co-creators and not merely receivers of knowledge. Four stories were created based on in-depth interviews conducted with community members (by DH, KL, LS) in the pilot village over 4-weeks, followed by a period of story outline writing consolidation and consultations with clinical researchers and academics. The implementation of an 8-week community-researcher filming project involved local villages as cast members, spectators, and location scouts. In some instances, some actors played themselves in the films.

The stories included: (i) Diriya Doni, a family story of a father/husband who is a dependent drinker, (ii) Aayubo Aawada, a story of a young man who loses his job and begins drinking to take away his worry, (iii) Apooru Iskole, a story about the harms of drinking from the perspective of school children whose parents drink, and (iv) Ekamuthu Gama, a story about how a community can work together to address corruption, alcohol production and distribution. Focus of the EE messages was to model what individuals, families, and communities can do to address alcohol-related harms; for example (i) three sisters, their mother and grandmother gain control of the family’s finances and pride and assist their father with his withdrawal from heavy alcohol use, (ii) a young man reflects on his past mistakes and decides to control his own harmful drinking habits and create a better life for himself, (iii) a school community, doctor, and social worker collect data regarding drinking habits from local people in their village, run an intervention program and relay health information back to drinkers in the village, (iv) a youth group and women’s group rally support from concerned citizens, facilitate community group meetings, run a public health campaign, create a series of street theatre shows dealing with themes of local alcohol use, track down and destroy a nearby brewing site, and visit police headquarters in the city to help stamp out local police corruption. These central protagonists reflect some of the real-world struggles of involved community members. Further details of the EE messages contained in each story are provided in the supplementary material.

**Figure 3.**
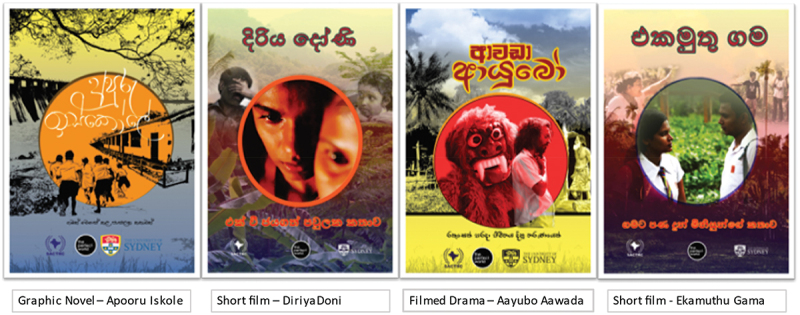
The THEATRE intervention EE materials – cover designs.

While the content of the stories was retained, in consultation with community members, the intervention EE components were converted (Figure 3). Specifically, stories (i) and (iv) were made into short films. The school story (iii) was adapted into a graphic novel, which are commonly read in rural Sri Lanka. The school story was selected for its likely appeal to resonate across multiple generations in a household. The story of the young man (ii) was written while in lockdown by NGA and DH and made into a filmed drama, which included traditional folk theatre (Sanni) elements [44], and elements from the original interviews conducted with the pilot village.

#### Health clinics – screening

Another component of the intervention is to provide individuals access to free health clinics, including screening for alcohol use and depression, and a brief intervention tailored to screening responses (see Pearson et al. for more information [32]). Two screening tools will be used: A culturally, contextually, and linguistically adapted version of the Alcohol Use Disorders Identification Test (AUDIT), developed by the World Health Organization to detect self-reported harmful or hazardous alcohol consumption [45]; and a validated version of the PHQ9 to assess depression [46]. In relation to alcohol, previous studies have shown screening linked to brief interventions can help reduce harmful or hazardous consumption after one year [47]. Originally, we intended to provide access to health screening prior to delivery of EE interventions, but this was not possible due to COVID-19. Instead, clinics are replaced by household visits by trained research staff to undertake screening assessments.

#### Alcohol brief interventions for harmful use of alcohol

An alcohol brief intervention will be offered if required following the screening assessment. Individuals are referred to treatment in cases where the AUDIT score is ≥7 (see Pearson et al. for more information [32]). Alcohol brief intervention is a short treatment strategy offered to prompt reflection on an individual’s thinking. It is based on motivational interviewing and uses techniques from the FRAMES approach [48]. Such alcohol brief interventions lie along the continuum of responses, from primary prevention to intensive treatment for individuals with severe alcohol use disorders. They have been found useful in middle-income settings, though rarely reported outside HICs [49]. An essential component of motivational interviewing is that the counsellor, in this case, a trained research assistant, has an empathetic approach, and focuses on the participant’s responsibility and choices and on enhancing the participant’s self-efficacy for change [50]. Originally, it was planned for medical staff to deliver the alcohol brief intervention following the screening. Due to limits in medical staffing, a senior clinician will train research staff (lay persons) to deliver brief interventions during household visits.

#### Public health messages

To distribute public health messages about harmful alcohol use, rational forms of persuasion will also be used. This includes using statistics and facts about alcohol-related harm [30]. Local health promotion students and faculty will develop related information materials at collaborating universities. These rational health messages about the harms of alcohol and the gains of moderating drinking will be distributed in the clusters at the time of the implementation of EE components.

#### Community advocates

Adaptations to the mode of delivery of screening clinics, EE components and participatory elements were necessary to avoid large social gatherings. Delivering a participatory community intervention remotely brings challenges, including planning and delivering the intervention, ensuring that intended participants receive the intervention without technical difficulties, and ensure engagement around the stories. To overcome this limitation, we will identify community members who will be trained to become ‘community advocates’ of the research project from within their communities. They are expected to fill the gap between the field research team and the community members and will have a key role in engaging people in discussions of the materials, running workshops, and collecting stories from the villages. For example, after EE stories are made available, village-level workshops will be conducted to enable individuals to share their stories through stories of hope. Community advocates will play a key role in this process.

There remain key uncertainties in the effectiveness of the materials, the delivery using digital formats, and the use of community advocates in this scaled-up intervention. There are also considerable differences in the communities selected that may help us to understand where the intervention is most suitable.

### Intervention implementation and evaluation

Whereas the baseline for this study was conducted in 20 villages, the intervention will be implemented in 15 villages, and rolled out in three steps [32]. Screening and brief intervention will be delivered in the first phase, followed by the four EE components delivered over a period of 12 weeks, facilitated by community advocates. A final household survey six months after completion of the intervention will collect individual, family, and community-level data on health, social capital, and financial stress data, complemented by an online survey of participants in the digital intervention and consented to online follow-up.

**Figure 4.**
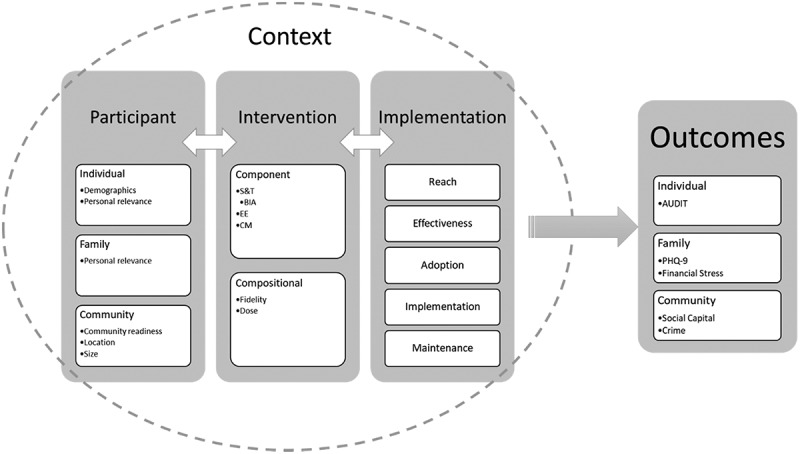
Design of evaluation of THEATRE study.

When designing the evaluation of a complex intervention, there is no consensus on the best approach. Increasingly, researchers are encouraged to utilise a range of perspectives, methodologies and outcomes for evaluating complex public health interventions [34,51]. The THEATRE study was designed with multiple interacting components, with several behaviours targeted at a number of levels, with several outcome variables. This requires an evaluation that reflects this complexity. We planned the evaluation to follow a modified process-outcome evaluation to include the relationships between the participants, the intervention, its implementation, and context using a mixed methods approach (Figure 4).

#### Evaluation relationships between participants and the intervention

The participant variables will include participant characteristics collected at baseline, a community readiness scale (five dimensions) [52], and the size and location of the village. In-depth interviews will be conducted to capture voices from study participants who participated in the intervention and those who did not. Interviews will be conducted in each village by trained research assistants using a thematic interview guide. The stories of hope, collected following the series of workshops, will also serve as data to evaluate the intervention.

The process evaluation of the intervention will include two parts: the individual components and the compositional effects. The alcohol brief interventions will be evaluated using a modified Attitudes to Psychological Interventions and Counselling (APIC-PC) [53] - a 13-item questionnaire to assess skills and knowledge, confidence to counsel, willingness to counsel and attitudes. The evaluation of EE components will include five elements related to the persuasiveness of the messages: health information recall, transportation, identification, systematic processing, and personal relevance [54] – all factors that have been associated with the effectiveness of EE [54]. The community mobilisation component of the intervention will be evaluated through a series of focus group discussions with community advocates and participants.

#### Evaluation of the relationship between the intervention and implementation

We will undertake an implementation analysis to include a range of measures based on the RE-AIM framework [27]. This framework focuses on five dimensions of implementation: Reach, Effectiveness, Adoption, Implementation and Maintenance. The effect of variation of implementation in villages forms a further unit of analysis. Details of the variables and data collection methods are included in Appendix 3. In addition, we measure specific aspects of the hybrid delivery. This will include online viewer metrics and digital confidence. The tools developed are available in the supplementary material.

#### Outcome data

Pre-and post-analyses will be conducted using combined and stratified (by online participation) individual-level data, and the proportion of each village participating digitally will be used as a confounder in analyses of aggregated village data. Measures include alcohol use (AUDIT), mental health (PHQ9), Social Capital (15 questions), Financial stress (4 questions) and health-related quality of life (EQ-5D). Further details of these measures and their collection is included in the study protocol [32].

#### Data analysis

To evaluate the ‘who, what, when, where and how’ of the effectiveness of the intervention we plan to employ deductive qualitative analysis [55]. This analysis involves three phases. In the first phase we will use the qualitative data of the participants to construct cases and their complexities. The second phase will include a comparison between the cases and the modified process and outcome evaluation. The third phase uses group analysis to discuss emerging findings from the first two phases. The group involved in the analysis will include researchers with different life experiences, training, and perspectives. Using the group to discuss emerging findings takes account of the complexity and variation of the social phenomena. The outcome of the final phase is the production of a hypothesis or set of hypotheses that fit the cases.

## Discussion

THEATRE is a complex, community-based intervention. It will evaluate whether a promising Sri Lankan pilot study that utilised arts-based research to moderate alcohol use can be scaled up. This protocol paper describes behaviour change theories, evaluation design, and modifications made to the study, which were needed as a result of the COVID-19 pandemic and the Sri Lankan financial crisis. Specifically, we present behaviour change theories with potential pathways to guide implementation and evaluation. Risky alcohol use is a public health challenge [1], influenced by social norms and stigma [56], and control and regulation are complicated in areas where the use of illicit alcohol is high [24]. In these settings, innovative approaches are needed to curb the harmful effects of alcohol use. As such, the intervention presented here is novel in the following ways:

The BCW functions, in combination with TDF, guided the design, development, and modifications of this study. This enabled a comprehensive framework to explore factors likely to influence behaviour change. Developed to identify the intervention components most appropriate to effect the desired change, the BCW has previously been used for intervention development and evaluation and to structure reviews – including in interventions focusing on alcohol [57,58]. For example, Gilchrist et al used the BCW during the theoretical development and feasibility testing of an intervention addressing substance use and intimate partner violence [59]. Of note, pre-specifying the behaviour change mechanisms in the design phase allowed us to, not only develop the intervention, but also to adapt it to the changing environment following COVID-19 and the financial crisis.

In recent years, the social determinants of mental health have achieved greater acknowledgement [60] as have approaches focusing on cultural and social determinants of mental health [61]. White and Sashidharan have called for global mental health interventions to focus on local context as a starting point for research, rather than as a consideration at the end of a study [62]. In keeping with this, the different components and modifications of our intervention were co-created and developed with community members. Here, we draw on traditional, Sri Lankan concepts and culture to strengthen delivery of messages, and ensure contextual appropriateness and acceptability. As outlined in this paper, involvement from community members will continue during intervention implementation, where they are provided with the opportunity to reflect on alcohol consumption.

If successful in moderating alcohol use, this arts-based, community-based intervention will be much-needed in improving well-being and reducing challenges from risky alcohol use for individuals, households, and communities. This will be important within Sri Lanka and beyond.

## Supplementary Material

Supplemental Material

## Data Availability

Deidentified data and code are available upon request (email: janebs@sund.ku.dk).

## Paper context

Multipronged interventions are needed to address the complex nature in which alcohol is consumed, including the use of illicit alcohol. Building on a promising pilot intervention that utilised arts-based efforts, this study will provide a deeper understanding of intervention components that makes it successful and relevant (or not). This paper presents the programme theory, intervention design, and modifications made due to multiple crises during the development and implementation phases.
