# Supplementary material for: Evaluating the programme and behavior change theories of a community alcohol education intervention in rural Sri Lanka: a study protocol
Source: Glob Health Action. 2023 Nov 16;16(1):2273625. doi: 10.1080/16549716.2023.2273625 (PMC10795635; doi:10.1080/16549716.2023.2273625)
Supplement: Supplemental Material [file ZGHA_A_2273625_SM7248.zip › Supplementary_file.docx]

*Supplementary file, Health messages in EE components*

| EE Component | Health communication target | Health message |
| --- | --- | --- |
| Apooru Iskole | Hope | Schools can support children, their families, and communities to support the impact of harmful use of alcohol |
|  | Availability of support | Key people in schools can provide a private space to listen to the students concerns.  Health and Education can work together to help address alcohol problems in the community  There are people in the community that can help families with their difficulties |
|  | Risk perception | Drinking can cause indirect problems for school children. |
|  | Positive behaviours | Schools can open a discussion with students about helping family members reduce their drinking.  School students have experiences of drinking in their homes and community.  Teachers and Principals can gather ideas of concerns to students.  Schools can gather information for a public health campaign |
|  | Social norms | Work with social worker to talk to groups in the community about harms of drinking |
|  | Empowerment | Working with partners in the community to participate in health checks and give advice on improving their health. |
| Diriya Doni | Hope | Change is possible even for heavy drinkers.  Your family can help |
|  | Availability of support | Dinali and her sisters help their father detox from alcohol.  Offers to support her father reduce his drinking |
|  | Risk perception | Reminds her father that the family need money for food.  Keep a mental note of his deteriorating health.  Confronts her father about spending the family’s money on kasippu and reminds him that they will now be without food. |
|  | Positive behaviours | Families can encourage their loved one to reduce drinking.  Dinali calls her mother who suggests alternative ways to ensure family has money for food.  Dinali discusses drinking once father is sober. |
|  | Social norms | Heavy drinking reduces the family ability to participate in important cultural events.  Heavy drinking impacts on important family events. |
|  | Empowerment | With family support you can reduce the harms from alcohol on you and your family. |
| Aayabo Aawada | Hope | Sometimes we make mistakes but there are always people willing to help you along the way. |
|  | Availability of support | Friends can provide you with someone to talk to about your problems. |
|  | Risk perception | Drinking can harm your ability to get or stay in a job.  There are always temptations to drink. |
|  | Positive behaviours | Talking to friends can help you understand your behaviour.  Cutting down your drinking can help you stay in your job.  Work can be a positive way to |
|  | Social norms | Drinking causes problems in how community sees you. |
|  | Empowerment | You have the power to banish the demon from your life. |
| Ekamuthu Gama | Hope | Working together as a community we can reduce the harms from alcohol. |
|  | Availability of support | Friends can support each other and share experiences of drinking in their household.  Gathering local people to discuss a plan to help their village.  The police can help the village address alcohol problems.  Sometimes a community may need to approach the next level of police officials in a nearby city to explain the problem if they do not get local support. |
|  | Risk perception | Moves away from danger when there is  a drinker in the house.  Identifying the sources of illegal alcohol in the community. |
|  | Positive behaviours | Talking in your community about the problems can be helpful.  Communities can make an action plan to address the problems.  A group of citizens can use creative tools (drama) to portray the harms of drinking.  Contacting police can help to get rid of illegal sales of alcohol. |
|  | Social norms | Problem drinking impacts people in many different ways.  Everyone should be given a chance to speak and share their perspective. |
|  | Empowerment | Working together in your community can address harmful use of alcohol.  Communities can work with Police to get rid of illegal alcohol outlets |

Health message recall

| Comic – school story | Film – family story | Drama – young person story | Film – community story |
| --- | --- | --- | --- |
| In the novel, *Rasika* attempts to reduce the harms of problem drinking for his students and their family members. What does he do?   - Asks for anonymous feedback from his students - Gathers the feedback and makes a plan of action - Offers to hear the students concerns in a private space - Enlists the help of a doctor - With the doctor, offers free health checks to members of the community - Gathers information for a public health campaign - Hires a social worker to talk to groups in the community about harms of drinking - Ensures the doctor relays results to people who participated in the health checks and give advice on improving their health - Opens a discussion with his students about helping family members reduce their drinking | During the film, *Dinali* decides to reduce the harms of problem drinking for her family. What does she do?   - Reminds her father that the family need money for food - Confronts her father about spending the family’s money on kasippu and reminds him that they will now be without food - Calls her mother who suggests she send any income she earns to their grandmother instead of her father - Keep a mental note of his deteriorating health - Encourages him to reduce his drinking - Offers to support her father reduce his drinking - Dinali and her sisters help their father detox from alcohol | In the filmed street theatre, *Nimal* attempts to reduce the harms of his own drinking. What does he do?   - Explains his problems to the demon - Seeks counsel with his friend - Begins to understand that drinking may have been the cause of some of his problems - Tries to reduce his drinking - Attempts to get a job and do a full day’s work - Banishes the demon from his life - Decides to not drink after work | During the film, *Nimesh* decides to reduce the harms of problem drinking in his village. What does he do?   - Moves away from danger when there is a drinker in the house - He identifies the local kasippu dealer and calls the police - He shares class notes with a fellow student (who lives with a drinker) - He gathers local people to discuss a plan to help their village. At that meeting, everyone is given a chance to speak and problem drinking is looked at from many perspectives - A group of citizens perform a play in their local village that portrays the harms of drinking - He gathers a group of young men, finds the brewing site, and destroys it - After discovering the local police are corrupt, he approaches the next level of police officials in a nearby city to explain the problem - He ensures the dealer is arrested |
